# ^13^C-metabolic flux analysis of respiratory chain disrupted strain Δ*ndhF1* of *Synechocystis* sp. PCC 6803

**DOI:** 10.1007/s12010-024-05138-4

**Published:** 2025-01-15

**Authors:** Keisuke Wada, Yoshihiro Toya, Fumio Matsuda, Hiroshi Shimizu

**Affiliations:** 1 Department of Bioinformatic Engineering, Graduate School of Information Science and Technology, Osaka University, 1-5 Yamadaoka, Suita, Osaka 565-0871 Japan; 2 Present Address: Research Institute for Sustainable Chemistry, National Institute of Industrial Advanced Science and Technology (AIST), Tsukuba Central 5-2, 1-1-1 Higashi, Tsukuba, Ibaraki 305-8565 Japan

**Keywords:** Photosynthesis, Respiration, *Synechocystis*, ^13^C-metabolic flux analysis, Cofactor balance, Cyclic electron transfer

## Abstract

Cyanobacteria are advantageous hosts for industrial applications toward achieving sustainable society due to their unique and superior properties such as atmospheric CO_2_ fixation via photosynthesis. However, cyanobacterial productivities tend to be weak compared to heterotrophic microbes. To enhance them, it is necessary to understand the fundamental metabolic mechanisms unique to cyanobacteria. In cyanobacteria, NADPH and ATP regenerated by linear and cyclic electron transfers using light energy are consumed by CO_2_ fixation in a central metabolic pathway. The previous study demonstrated that the strain deleted a part of respiratory chain complex (Δ*ndhF1*) perturbed NADPH levels and photosynthetic activity in *Synechocystis* sp. PCC 6803. It is expected that disruption of *ndhF1* would result in a decrease in the function of cyclic electron transfer, which controls the ATP/NAD(P)H production ratio properly. In this study, we evaluated the effects of *ndhF1* deletion on central metabolism and photosynthesis by ^13^C-metabolic flux analysis. As results of culturing the control and Δ*ndhF1* strains in a medium containing [1,2-^13^C] glucose and estimating the flux distribution, CO_2_ fixation rate by RuBisCO was decreased to be less than half in the Δ*ndhF1* strain. In addition, the regeneration rate of NAD(P)H and ATP by the photosystem, which can be estimated from the flux distribution, also decreased to be less than half in the Δ*ndhF1* strain, whereas no significant difference was observed in ATP/NAD(P)H production ratio between the control and the Δ*ndhF1* strains. Our result suggests that the ratio of utilization of cyclic electron transfer is not reduced in the Δ*ndhF1* strain unexpectedly.

**Supplementary Information:**

The online version contains supplementary material available at 10.1007/s12010-024-05138-4.

## Introduction

Since cyanobacteria can grow using light energy and atmospheric CO_2_, it is attracting attention as a host for producing chemicals from CO_2_ toward achieving a sustainable society [1]. Cyanobacteria possess several advantageous properties for industrial applications: (i) atmospheric CO_2_ fixation as a sole carbon source by RuBisCO, (ii) low contamination risks due to quick growth on simple media, and (iii) higher photosynthetic efficiency than land plant [2]. Previous studies successfully produced various valuable compounds, including polyhydroxyalkanoates [3], alcohols [4, 5], carbohydrates [6–8], organic acids [9, 10], and isoprene derivatives [11–13], using genetically engineered cyanobacterial strains. However, productivities tend to be weak compared to other microbes that can utilize higher energy contents such as sugars and oils. To enhance the cyanobacterial productions, it is necessary to understand the cyanobacteria-specific functional connection between photosystem producing the energy required to fix CO_2_ and central metabolic pathway responsible for the conversion from the CO_2_ to the target products.

**Fig. 1:**
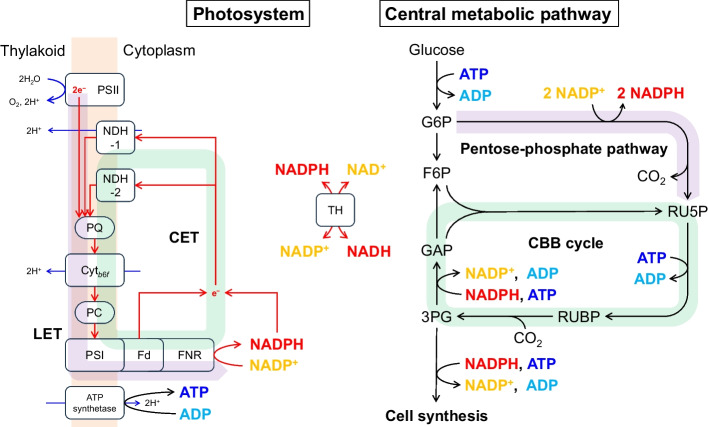
Relationship between photosystem and central metabolic pathway in cyanobacteria connected by NAD(P)H and ATP. In linear electron transfer, electrons extracted from water in photosystem II (PSII) are transferred to photosystem I (PSI) via cytochrome (Cyt *b*_6_*f*) and used for NAD(P)H regeneration. ATP is also regenerated using protons (H^+^) pumped out during the electron transfer process. In cyclic electron transfer, electrons from ferredoxin (Fd) or NAD(P)H are transferred to plastoquinone (PQ), thereby regenerating ATP without regenerating NAD(P)H. Transhydrogenase (TH) easily converted electron transfer between NADH and NADPH. CO_2_ is fixed by RuBisCO in central metabolic pathway using NAD(P)H and ATP produced by the photosystem

In case of cyanobacterial photosynthesis, the photosystem produces NADPH and ATP, which are used in a sequence of reactions involving CO_2_ fixation by the RuBisCO, as known as Calvin-Benson-Bessham (CBB) cycle. The relationship between linear electron transfer (LET) and cyclic electron transfer (CET), which are typical electron flows in photosystems [14], and the CBB cycle is shown in Fig. 1. In the LET, electrons extracted from water in photosystem II (PSII) are transferred to NADP^+^ via plastoquinone (PQ) followed by the cytochrome *b*_6_*f* complex (Cyt *b*_6_*f*), plastocyanin (PC), and photosystem I (PSI). Transhydrogenase (TH) converts NADPH with NAD^+^ to NADP^+^ with NADH and vice versa. The LET produces NADPH and ATP in a molar ratio 2:2.57, whereas the molar ratio of NADPH and ATP consumed in the CBB cycle is 2:3. To address this ATP shortage in the LET, cyanobacteria utilize another electron transfer system, CET. The CET produce ATP without NADPH production by transferring electron from ferredoxin (Fd) to PQ for reacting to fluctuating environmental conditions [15, 16]. One of the electron acceptors in CET is NAD(P)H-dehydrogenase I (NDH-1) as respiratory chain complex. Toyoshima et al. revealed that NDH-1 was utilized under favorable growth conditions [17]. Battchikova et al. revealed that deletion of *ndhS*, which constitutes a new subunit of NDH-1, triggered reduction of growth rate and CET activity mediated by the NDH-1 [18]. These results suggest that the NDH-1 is a pivotal electron acceptor in CET. Hence, it is considered that NDH-1 dysfunction can perturb the link between photosystem and central metabolic pathway via NAD(P)H.

According to updated-Fluorome summarizing the chlorophyll fluorescence of 750 gene-disruptant from *Synechocystis* sp. PCC 6803, the strain disrupted *ndhF1* encoding NAD(P)H-quinone oxidoreductase subunit 5 in NDH-1 (Δ*ndhF1*) showed the largest change in the Kautsky curve [19]. Although it has been shown that the photosynthetic activity estimated from chlorophyll fluorescence in mutants with defective respiration, including the Δ*ndhF1* strain, may be increased [20], the NADPH accumulated in the dark [21]. Since deletion of genes regarding NDH-1 resulted in the accumulation of excess NAD(P)H available for biosynthesis of target products, NDH-1 dysfunction may be effective for productions of valuable compounds in cyanobacteria. In facts, deletion of *ndhF1* in engineered-cyanobacterial strains triggered increase of the production of ethanol and 1,3-propandiol, which consumed NAD(P)H for biosynthesis, via expansion of NAD(P)H sink [22, 23]. Therefore, it is expected that the analysis of the Δ*ndhF1* strain without producing the NAD(P)H-consuming chemical productions is useful for understanding the fundamental metabolism of cyanobacteria about NAD(P)H perturbation.

Although the regenerative fluxes of NAPDH and ATP by photosystem cannot be measured directly, the balance between regeneration and consumption rates of them in cyanobacteria is maintained homeostasis by the photosystem and central metabolism. Therefore, by determining the regeneration and consumption rates of them by the central metabolic pathway, it is possible to estimate those by photosynthesis. ^13^C-metabolic flux analysis (^13^C-MFA) is a method that accurately predicts the flux distribution of metabolic pathway, including the CO_2_ fixation rate, using isotope labeling information [24]. One of the insights from ^13^C-MFA is an estimation of the intracellular state based on cumulation of the regeneration and consumption rates of NAD(P)H and ATP accompanying metabolic reactions [15, 25]. In this study, we aimed to investigate how the metabolic state changes in the Δ*ndhF1* strain by ^13^C-MFA. We cultivated the Δ*ndhF1* strain in medium containing glucose and carbonate, estimated the flux distribution on the central metabolic pathway during the exponential growth phase, and evaluated the effects of NDH-1 dysfunction on the overall metabolism of cyanobacteria.

## Materials and Methods

### Bacterial Strains and Culture Condition

The glucose-tolerant strain of *Synechocystis* sp. PCC 6803 (Ctrl) and its derivative strain lacking *ndhF1* (*slr0844*) encoding NAD(P)H-quinone oxidoreductase subunit 5 (Δ*ndhF1*) [20] used in this study were kindly gifted by Prof. Kintake Sonoike. Both strains were grown on modified BG-11 (1.5 g L^−1^ NaNO_3_, 0.027 g L^−1^ CaCl_2_·12H_2_O, 4.5 mg L^−1^ FeCl_2_·4H_2_O, 1.2 mg L^−1^ NH_4_Cl, 1 mg L^−1^ EDTA-2Na, 31 mg L^−1^ K_2_HPO_4_, 75 mg L^−1^ MgSO_4_·7H_2_O, 2.9 mg L^−1^ H_3_BO_4_, 1.8 mg L^−1^ MnCl_2_·4H_2_O, 0.22 mg L^−1^ ZnSO_4_·7H_2_O, 0.39 mg L^−1^ Na_2_MoO_4_·2H_2_O, 79 μg L^−1^ CuSO_4_·5H_2_O, and 49 μg L^−1^ CoCl_2_·6H_2_O, and 4.8 g L^−1^ HEPES–KOH (pH 7.5)). Cultures were performed four biological replicates. The solid medium contained 15 g L^−1^ agar. 0.02 g L^−1^ erythromycin, 0.9 g L^−1^ (5 mM) [1,2-^13^C] glucose, and 4.2 g L^−1^ (50 mM) NaHCO_3_ were added whenever necessary. [1,2-^13^C] glucose was purchased from Cambridge Isotope Laboratories (Andover, MA, USA). The intensity of the light illumination was measured with a photometer LI-250A (LI-COR Inc.; Lincoln, NE, USA) containing a quantum sensor LI-190SA (LI-COR Inc.).

For pre-culture, both strains were cultured in 20 mL of BG-11 medium containing NaHCO_3_ in 100-mL Erlenmeyer flasks under fluorescent light flux of 100 μmol m^−2^ s^−1^ light at 30℃. Cell growth was monitored by measuring optical density at 730 nm (OD_730_) with the UVmini-1240 system (Shimadzu; Kyoto, Japan). When OD_730_ ≈ 1, pre-cultures were inoculated in 100 mL of BG-11 medium containing [1,2-^13^C] glucose and NaHCO_3_ in 500-mL Erlenmeyer flask as the main culture under fluorescent light flux of 50 μmol m^−2^ s^−1^ light with an initial OD_730_ of 0.05.

### Measurements of Dry Cell Weight, Extracellular Glucose, Intracellular Chlorophyll, and Whole Cell Absorption Spectra

To calculate the coefficient of dry cell weight (DCW) per L per OD_730_, 25 mL culture broth were filtered with OD_730_ ≈ 1 using 0.2 μm pore size Omnipore filter disks (Merck KGaA; Darmstadt, Germany). The cells on filter were washed with 0.9% NaCl, dried at 70℃. The dried cells were weighed together with the filter. To exclude the weight of filter, 25 mL culture broth without microbes was processed in a same procedure and the result was set as a control.

To measure glucose concentrations in culture broth, culture broth was collected every time points and its supernatant was filtrated using a 0.45-μm pore size Millex HV filter (Merck KGaA). The glucose concentrations in filtrates were measured with an enzymatic electrode sensor BF-5 (Oji Scientific Instruments; Hyogo, Japan).

To measure intracellular chlorophyll concentration, 2 mL culture broth was collected with OD_730_ ≈ 2 and extracted chlorophyll with methanol according to the previous study [26, 27]. Extracted chlorophyll was measured using a Synergy HTX (BioTek; Winooski, VT, USA) at 665 and 750 nm wavelengths.

To measure whole cell absorption spectra, culture broth with OD_730_ ≈ 2 was measured at room temperature with 1-nm increments using a spectrophotometer DU800 (Beckman Coulter; Fullerton, CA, USA). Absorption spectra were normalized to turbidity measured at 750 nm.

### GC/MS for Labeling Pattern of Proteinogenic Amino Acids

The method extraction and analysis of proteinogenic amino acid basically followed the previous study [28]. Ten mL of quadruplicate culture of the control and the engineered strains were taken from the flask, centrifuged at 7000 × *g* for 5 min at 4℃, and hydrolyzed in 6 mol L^−1^ HCl at 105℃ for 18 h. The resulting proteinogenic amino acids were derivatized with *N*-(*tert*-butyldimethylsilyl)-*N*-methyl-trifluoroacetamide containing *tert*-butyldimethylchlorosilane in acetonitrile at 105℃ for 1 h and then analyzed using a gas chromatograph—mass spectrometer (GC–MS; 7890A, 5975C; Agilent Technologies; Santa Clara, USA) equipped with a DB-5MS + DG column (Agilent Technologies). The analytical conditions used are described elsewhere [29]. The data obtained from GC–MS were corrected by reduction of the natural abundance ratio of C, H, O, N, and Si isotopes according to the previous study [30].

### ^*13*^*C-Metabolic Flux Analysis*

^13^C-MFA was performed using in-house software OpenMebius [31], which is based on the elementary metabolite units framework [32] in MATLAB (MathWorks Inc.; Natick, MA, USA). The biomass composition of *Synechocystis* sp. PCC 6803 described in previous study [33], and specific growth rates measured in this study were employed for estimating biomass synthesis fluxes for each strain. The metabolic pathway model of *Synechocystis* sp. PCC 6803 comprised 40 reactions containing CBB cycle, oxidative pentose-phosphate pathway, glycolysis, anaplerotic reaction, Krebs cycle, and glyoxylate shunt (Supplementary Table 1). Metabolic fluxes were estimated by minimizing the residual sum of squares between the experimentally measured and model predicted ^13^C-enrichment using the *fmincon* optimization solver in the MATLAB toolbox. The standard deviation of measured ^13^C-enrichment was set to 0.01 [28]. The specific rates of growth and glucose consumption were used as measurable fluxes. The optimizing function is described as

$$Minimize RSS= \sum_{i=1}^{n}{\left(\frac{{MID}_{i}^{measured}-{MID}_{i}^{simulated}}{{SD}_{i}}\right)}^{2}+\sum_{j=1}^m{\left(\frac{{r}_{j}^{measured}-{r}_{j}^{simulated}}{{SD}_{j}}\right)}^{2}$$

where $n$ and $m$ represent the number of amino acids and measurable fluxes used for flux estimation, respectively; ${MID}_{i}^{measured}$ and ${MID}_{i}^{simulated}$ represent the mass isotopomer distribution (MID) of $i$-th measured and estimated amino acids, respectively; ${SD}_{i}$ and ${SD}_{j}$ represent the SD of $j$-th measured MID and measured fluxes, respectively; and ${r}_{j}^{measured}$ and ${r}_{j}^{simulated}$ are $j$-th measured and estimated fluxes, respectively.

## Results

### Phenotypical Profiles

The *ndhF1* encodes NAD(P)H-quinone oxidoreductase chain 5, which forms proton motive force as respiratory electron transfer using NAD(P)H as a reducing power. Ogawa et al. constructed a mutant strain, Δ*ndhF1*, and revealed that the dysfunction of NDH-1, which is the main electron acceptor in CET, resulted in significant alteration in photosynthetic activity [20]. To evaluate in detail the alterations in central metabolism in response to the alterations in phenotypes caused by *ndhF1* disruption, we used the Ctrl and Δ*ndhF1* strains in this study.

**Fig. 2:**
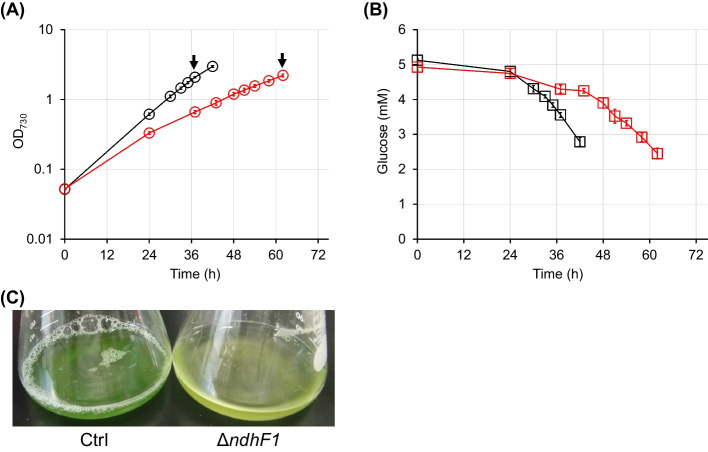
Phenotypical profiles of the Ctrl (white) and Δ*ndhF1* (red) strains grown in modified BG-11 containing [1,2-^13^C] glucose under fluorescent light with 50 μmol m^−2^ s^−1^ (mean ± SD, *N* = 4). **A** Growth profiles. Arrows indicate a sampling point for ^13^C-MFA. **B** Glucose consumption profiles. **C** Culture broth at a sampling point for ^13^C-MFA

The Ctrl and Δ*ndhF1* strains were cultured in modified BG-11 medium containing [1,2-^13^C] glucose and NaHCO_3_ as carbon sources under fluorescent lights with 50 μmol m^−2^ s^−1^ light. The growth characteristics are shown in Fig. 2. The specific rates of growth and glucose consumption at exponential growth phase (Ctrl; 30–37 h, Δ*ndhF1*; 48–58 h) and chlorophyll contents at a sampling point for ^13^C-MFA (Ctrl; 37 h, Δ*ndhF1*; 58 h) were summarized in Table 1.

**Table 1:** Growth characteristics of the Ctrl and Δ*ndhF1* strains

Strain	Specific growth rate (h^−1^)	Specific glucose consumption rate (mmol gDCW^−1^ h^−1^)	Chlorophyll contents (mg gDCW^−1^)
Ctrl	0.090 ± 0.006	0.232 ± 0.037	16.31 ± 0.47
Δ*ndhF1*	0.044 ± 0.002	0.210 ± 0.022	5.72 ± 0.29

Specific growth and glucose consumption rates were calculated in exponential growth phase with OD_730_ ≈ 2 (Ctrl; 30–37 h, Δ*ndhF1*; 48–51 h). Data shown are mean ± SD (*N* = 4)

Regarding the growth profiles, the Δ*ndhF1* strain required additional time to reach the same OD_730_ compared to the Ctrl strain (Fig. 2A). This phenotype was a typical in NDH-1 deficient strain and consisted with the results in the previous studies [18, 34, 35]. Regarding the glucose consumption profiles, the slow-growing Δ*ndhF1* strain consumed glucose more slowly than the Ctrl strain (Fig. 2B). Cells of the Δ*ndhF1* strain were paler green than those of the Ctrl strain (Fig. 2C), implying decrease of chlorophyll contents in cells. The chlorophyll content in the Δ*ndhF1* strain was certainly decreased to 35% (= 5.72/16.31) of that in the Ctrl strain (Table 1). The results of UV–VIS absorbance spectra of whole intact cells showed that the peak height of phycocyanin (620 nm) per chlorophyll α (680 nm) for the Δ*ndhF1* strain was smaller than for the Ctrl strain (Supplementary Fig. 1). This phenomenon, which may be caused by glucose, was more dramatic than previous study [20]. It is expected that the photosynthetic activity of the Δ*ndhF1* strain was weaker than that of the Ctrl strain. In fact, the specific rates of glucose consumption and growth of the *ΔndhF1* strain were also reduced to 68% (= 0.21/0.31) and 44% (= 0.04/0.09) of the Ctrl strain, respectively (Table 1). These results suggest that cellular activities, including energy production and CO_2_ fixation, were decreased in the Δ*ndhF1* strain by NDH-1 dysfunction.

### *Analyses for *^*13*^*C-Labelling Pattern in Proteinogenic Amino Acids*

Proteinogenic amino acids contained in cells of the Ctrl and Δ*ndhF1* strains in exponential growth phase (OD_730_ ≈ 1) were analyzed using a GC–MS to obtain raw MID. Noise derived from natural isotopes contained in raw MID was removed.

**Fig. 3:**
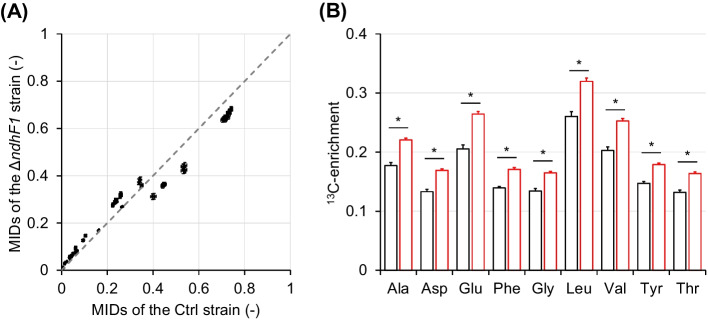
Comparison of ^13^C-labelled proteinogenic amino acids in the Ctrl and Δ*ndhF1* strains (mean ± SD, *N* = 4). **A** Scatter plot for MIDs of proteinogenic amino acid of the Ctrl and Δ*ndhF1* strains. **B** Comparison of ^13^C-enrichment for each proteinogenic amino acid. ^13^C-enrichment was determined by calculating the proportion of ^13^C-labeled carbon atoms among the skeleton carbon for the [M-57]^+^ fragment. Leu fragment showed [M-159]^+^ fragment because its [M-57]^+^ fragment cannot be measured. White; Ctrl, red; Δ*ndhF1*. Asterisks indicate *p* < 0.0001 in Student’s *t*-test

A scatter plot of MIDs of the Ctrl and Δ*ndhF1* strains is shown in Fig. 3A. Some MIDs were plotted off a diagonal, indicating that the proteinogenic amino acid labeling pattern changes between strains. To examine this in more detail, we calculated the proportion of ^13^C-labeled carbon atom (^13^C-enrichment) among the backbone carbon atoms of each amino acid (Fig. 3B). For all amino acids, the ^13^C-enrichment in the Δ*ndhF1* strain was greater than that in the Ctrl strain. To increase the ^13^C-labeled cellular component in the Δ*ndhF1* strain, it is necessary to incorporate more ^13^C-labeled carbon from glucose into the cellular components than carbon from unlabeled CO_2_ fixed by the RuBisCO, etc. It was expected that the CO_2_ fixation rate of the Δ*ndhF1* strain was lower than that of the Ctrl strain.

### Flux Distribution of Central Metabolic Pathway

**Fig. 4:**
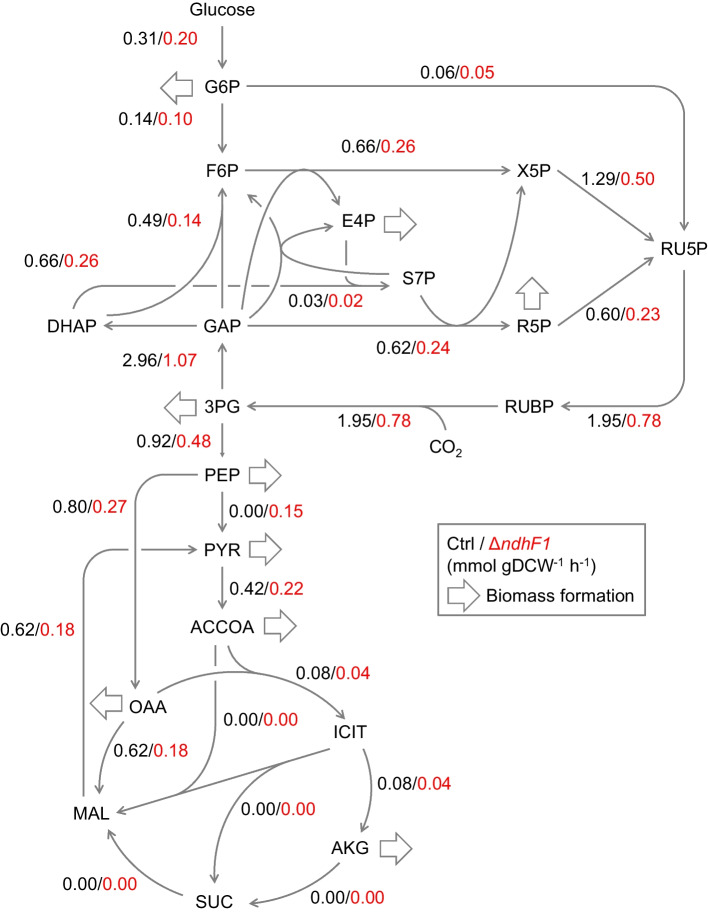
Metabolic flux distributions in the central metabolic pathways of the Ctrl and Δ*ndhF1* strains during exponential growth phase. The arrows between metabolites the direction of the reaction. The numbers accompanying the arrows indicate best-fit values of absolute metabolic fluxes. The large arrows indicate biomass formation fluxes required for cell synthesis determined from the specific growth rates

^13^C-MFA was performed using the MID of proteinogenic amino acids and the specific rates shown in Table 1. A flux distribution that can explain both the actual MID and specific rates was estimated by optimization calculations. The metabolic flux (mmol gDCW^−1^ h^−1^) calculated as the best-fit value for each reaction is shown in Fig. 4. The measured and estimated MID values are shown in Supplementary Table 2. Since metabolic model has 20 degrees of freedom and the MID and specific rates used for optimization have 122 degrees of freedom, the threshold for the chi-squared test is 126.5. The residual sum of squares of the MID calculated from the estimated flux distribution and the measured MID for the Ctrl and Δ*ndhF1* strains were 90.3 and 124.6. Since the residual sum of squares value of the both strains passed the chi-squared test, the estimated flux distribution can be regarded as statistically identical to the actual flux distribution.

The rate of CO_2_ fixation by RuBisCO in the Ctrl strain was comparable to that in the previous study [36], demonstrating the validity of the results of this analysis. The CO_2_ fixation rate in the Δ*ndhF1* strain was reduced to 40% (= 0.78/1.95) of that in the Ctrl strain, which was also consistent with the results expected from the phenotypical profile and MID. Accompanied with the decrease of CO_2_ fixation rate, the fluxes of the entire pathways also decreased in the Δ*ndhF1* strain. However, almost no difference was observed in the flux ratio at the branch points of the metabolic pathway; 76% (= 2.96/(1.95×2)) and 69% (= 1.07/(0.78×2)) of rates of CO_2_ fixation by RuBisCO redistributed to CBB cycle in the Ctrl and Δ*ndhF1* strains, respectively. The fluxes to the pentose-phosphate pathway of the Ctrl and Δ*ndhF1* strains were 0.06 and 0.05 mmol gDCW^−1^ h^−1^, respectively, indicating that NADPH regeneration by pentose-phosphate pathway was small at least during the exponential growth phase even under glucose presence conditions.

### *Estimation of Photosynthetic Activities from Cofactor Balance Revealed by *^*13*^*C-MFA*

Fluxes on photosystem cannot measure directly. However, it is considered that the mass balance of coenzymes, including NADPH and ATP, are satisfied in whole cell level to maintain homeostasis in a pseudo-steady state such as the exponential growth phase. In cyanobacteria, NADPH and ATP were produced by central metabolic pathway, but also by photosystem. It is expected that the imbalance between regeneration and consumption of the coenzymes by central metabolic pathway were compensated by photosystem. Therefore, it is possible to indirectly estimate the fluxes of the photosystem by calculating the imbalance of coenzymes between regeneration and consumption by the central metabolism [15, 25]. It was assumed that there was no distinction between NADH and NADPH due to the presence of TH in this study.

Since the *ndhF1* encodes a subunit of NDH-1 complex, it is inferred that the contribution of CET is reduced in the Δ*ndhF1* strain. If the ratio of CET to LET decreased, ATP regeneration relative to NAD(P)H regeneration by the photosystem also should decreased. Therefore, we evaluated the regeneration rates of NAD(P)H and ATP by the photosystem for the Ctrl and Δ*ndhF1* strains based on flux distribution.

**Fig. 5:**
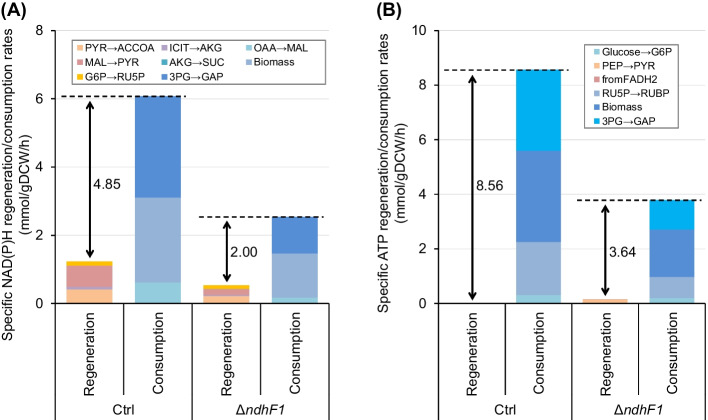
The total NAD(P)H (**A**) and ATP (**B**) regeneration/consumption rates estimated from best-fit values of absolute metabolic fluxes in the Ctrl and *ΔndhF1* strains. The lacking NADPH and ATP regeneration fluxes (arrows) should be equilibrated by photosynthesis

Fluxes related to regeneration and consumption of NAD(P)H and ATP were cumulated (Fig. 5). In both strains, consumption of both NAD(P)H and ATP exceeded regeneration. The regeneration fluxes of NAD(P)H and ATP by photosystem estimated from the gap between regeneration and consumption in NAD(P)H and ATP by central metabolic pathway in the Ctrl strain were 4.85 and 8.56 mmol gDCW^−1^ h^−1^, whereas those in the Δ*ndhF1* strain were 2.00 and 3.64 mmol gDCW^−1^ h^−1^, respectively. These results suggest that NADPH and ATP in both strains produced almost exclusively by photosystems. NADPH derived from NADH regenerated by pyruvate dehydrogenase (PYR → ACCOA) per total NADPH regeneration in the Ctrl and Δ*ndhF1* strain were 7% (= 0.42/6.08) and 9% (= 0.22/2.54), respectively, suggesting contribution of NADH was quite low for source of reducing power than NADPH. The estimated regeneration fluxes of both coenzymes also decreased in the Δ*ndhF1* strain, but the reduction ratios were 41% (= 2.00/4.85) and 43% (= 3.64/8.56), indicating that there was almost no difference between both strains. If CET utilization ratio increase, ATP/NAD(P)H production ratio also increase since ATP is produced without NADPH production. However, the ATP/NAD(P)H production ratio of the Ctrl and Δ*ndhF1* strain were 1.77 (= 8.56/4.85) and 1.82 (= 3.64/2.00), respectively, suggesting that there was no obvious difference between both strains, too. Yamamoto et al. demonstrated that the ATP/NAD(P)H production ratio reflected the utilization ratio of CET/LET ratio [15]. These results suggest that the utilization ratio of CET remains unchanged under dysfunction of NDH-1 which contribute as a major electron acceptor in CET.

## Discussion

In this study, we evaluated metabolic states of the Δ*ndhF1* and Ctrl strains by ^13^C-MFA. The specific rates of growth and glucose consumption, and contents of photosynthetic pigments in the Δ*ndhF1* were decreased compared to the Ctrl strain (Fig. 2, Table 1, Supplementary Fig. 1). Accompanied with the reduction of them, the CO_2_ fixation rate by RuBisCO in the Δ*ndhF1* also decreased compared to the Ctrl strain, whereas no obvious change of flux ratio at the branch points in metabolic pathway was observed (Fig. 4). Although the total specific NAD(P)H and ATP regeneration/consumption rates were decreased in the Δ*ndhF1*, regenerative NAD(P)H/ATP ratio by photosynthesis was comparable between the Ctrl and Δ*ndhF1* strains (Fig. 5). The previous study demonstrated that the change of ATP/NAD(P)H production ratio reflected utilization ratio of CET/LET ratio [15]. These results suggest that the total photosynthetic activities estimated from ^13^C-MFA decrease under NDH-1 dysfunction, but the utilization ratio of CET remains unchanged against expectations.

Despite the disruption of *ndhF1*, which constituted a main electron acceptor NDH-1 in CET, no obvious change was observed in the utilization ratio of CET. The fact that the CET utilization ratio was stably maintained may indicate that this level of CET utilization ratio was necessary under the culture conditions used in this study. According to the previous study [15], the CET/LET ration in *Synechocystis* sp. PCC 6803 under mixotrophic condition under 3 kinds of single wavelength lights was estimated to 0.2–2.2. Of these, the growth rate, flux distribution, and ATP/NADPH ratio in this study were middle level between R630 and R680, which have CET/LET rations of 0.2–0.5 and 0.5–1.0, respectively. Hence, it is expected that CET/LET rations of 0.2–0.5 were required for growth of *Synechocystis* sp. PCC 6803 under mixotrophic conditions. Since CET is a pivotal system to produce ATP without NADPH regeneration for responding flexibly to changing environments, there are some alternative pathways [37]. NAD(P)H dehydrogenase II (NDH-2) is a one of the candidates for alternative electron acceptors. NDH-2, unlike NDH-1, reduces PQ without formation of proton gradient during the electron transfer via itself [38]. Although the efficiency of ATP production via the oxidation of NAD(P)H by NDH-2 is smaller than that by NDH-1, its function may be advantageous under NAD(P)H-rich conditions such as *ndhF1* deletion, since excess NAD(P)H can be consumed without futile ATP production. Although a few reports showed the functionality of NDH-2 in *Synechocystis* sp. PCC 6803 [39, 40], these were insufficient to estimate the degree of contribution to CET by NDH-2. Another candidate for electron acceptors in CET is a proton gradient regulation 5 (PGR5) coupled with PGR5-like photosynthetic phenotype 1 (PGRL1). PGR5/PGRL1 transfers electron from Fd to PQ directly or indirectly [41]. The previous studies reported that their analogs were found in *Synechocystis* sp. PCC 6803 genome and stimulated CET as electron acceptors [42, 43]. However, the CET efficiency using alternative electron acceptors seemed to be much lower than that using NDH-1, since parameters related to cyanobacterial growth decreased across the board in this study, especially photosynthetic pigments. PC localizes in phycobilisome for harvesting light energy, while chlorophyll localizes in PSII and PSI for transferring light energy. Since these pigments contributes to efficient photosynthetic activities, decrease of these pigments should be effective to avoid production of the excess NAD(P)H in absence of *ndhF1* in exchange for well-growth.

As shown in the previous studies for enhancing productivities of target products which required NAD(P)H for biosynthesis, it is certainly a great idea in which *ndhF1* disruption for making excess available NAD(P)H pool in engineered-cyanobacterial strains [22, 23]. However, this study revealed that *ndhF1* disruption also triggered some unfavorable decreases in cyanobacterial metabolism, including growth rate, photosynthetic pigment contents, and CO_2_ fixation rate. It may be caused by the excess NAD(P)H pool. One solution is to consume excess NAD(P)H accumulated by the disruption of *ndhF1* to an appropriate level by enhancing the productions of target compounds requiring NAD(P)H for their biosynthesis using genetic engineering techniques. Although this strategy is expected to improve cyanobacterial growth to some extent, the carbon shortage will limit whole cyanobacterial metabolism because of current state that ca. 70% of CO_2_ fixation rate must be redistributed into the CBB cycle to maintain the current CO_2_ fixation rate. Hence, most important improvement for cyanobacterial metabolism is enhancement of efficiency of CO_2_ fixation by RuBisCO via CBB cycle. Although it is a challenging issue, success for improvement will open up a new frontier for cyanobacterial production.

## Supplementary Information

Below is the link to the electronic supplementary material.

Supplementary file1 (XLSX 23 KB)

Supplementary file2 (PDF 16 KB)

## Data Availability

Not applicable.

## Abbreviations

G6P: Glucose 6-phosphate
F6P: Fructose 6-phosphate
GAP: Glyceraldehyde 3-phosphate
DHAP: Dihydroxyacetone phosphate
RU5P: Ribulose 5-phosphate
X5P: Xylulose 5-phosphate
R5P: Ribose 5-phosphate
S7P: Sedoheptulose 7-phosphate
E4P: Erythrose 4-phosphate
RUBP: Ribulose 1,5-phosphate
3PG: 3-Phosphoglycerate
PEP: Phosphoenolpyruvate
PYR: Pyruvate
ACCOA: Acetyl-CoA
ICIT: Isocitrate
AKG: α-Ketoglutarate
SUC: Succinate
MAL: Malate
OAA: Oxaloacetate
