# Supplementary figures and images for: 13C-metabolic flux analysis of respiratory chain disrupted strain ΔndhF1 of Synechocystis sp. PCC 6803
Source: Appl Biochem Biotechnol. 2025 Jan 15;197(5):2944–57. doi: 10.1007/s12010-024-05138-4 (PMC12065754; doi:10.1007/s12010-024-05138-4)

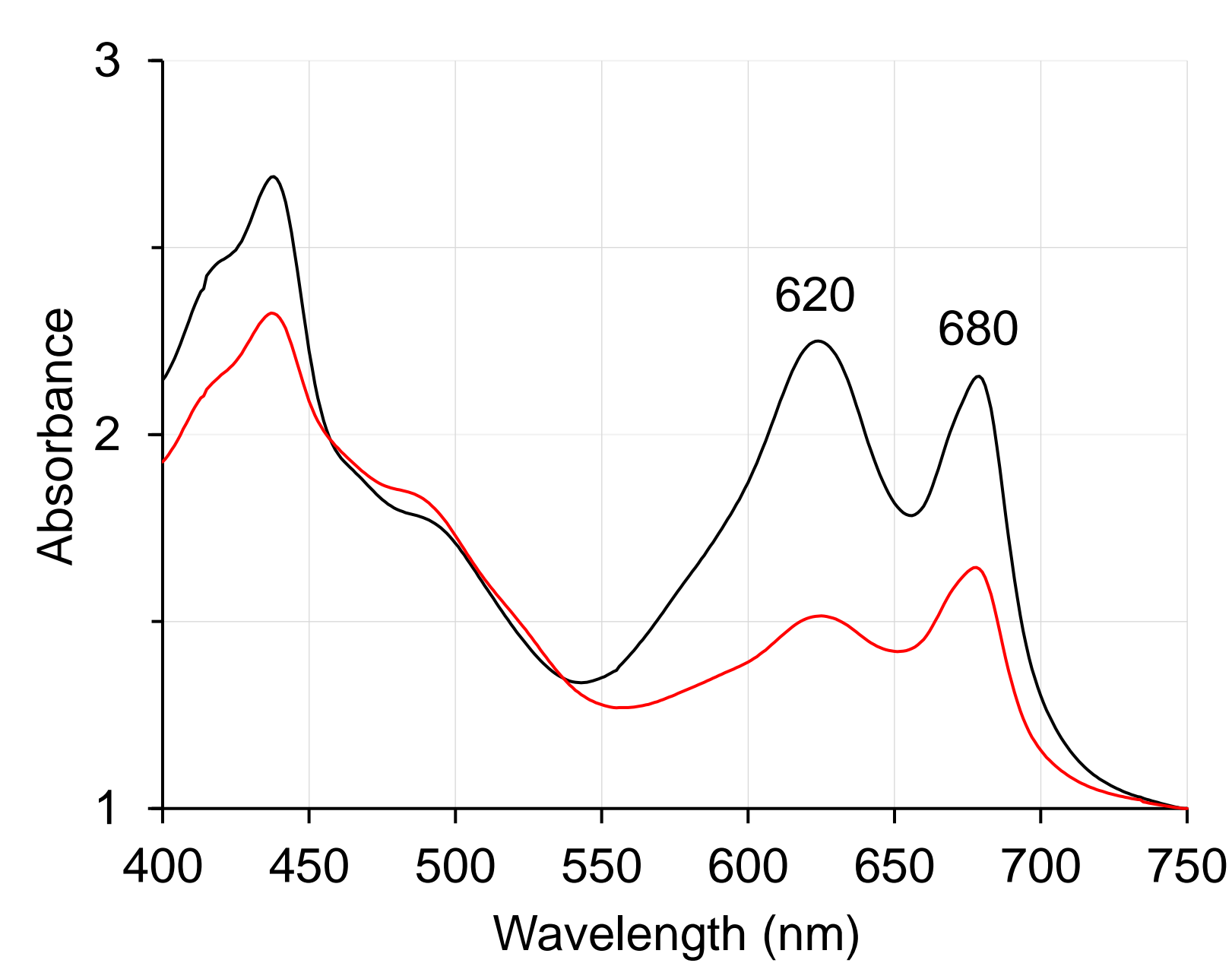

Supplement: Supplementary file 2 — Supplementary file2 (PDF 16 KB) [file 12010_2024_5138_MOESM2_ESM.pdf]
